# Arginase-1 Released into CSF After Aneurysmal Subarachnoid Hemorrhage Decreases Arginine/Ornithine Ratio: a Novel Prognostic Biomarker

**DOI:** 10.1007/s12975-021-00944-y

**Published:** 2021-10-02

**Authors:** Julian Zimmermann, Johannes Weller, Sven Grub, Sied Kebir, Felix Lehmann, Hartmut Vatter, Patrick Schuss, Erdem Güresir, Marcus Müller

**Affiliations:** 1 Department of Neurology, University Hospital Bonn, Venusberg-Campus 1, 53127 Bonn, Germany; 2 Department of Anaesthesiology and Intensive Care, University Hospital Bonn, Bonn, Germany; 3 Department of Neurosurgery, University Hospital Bonn, Bonn, Germany

**Keywords:** Subarachnoid hemorrhage, Cerebrospinal fluid, Vasospasm, Prognosis, NO-metabolism, Arginase

## Abstract

We hypothesized that the enzyme arginase-1 is released into the cerebrospinal fluid (CSF) during red blood cell lysis and contributes to dysregulated metabolism of the nitric oxide (NO) precursor L-arginine during aneurysmal subarachnoid hemorrhage (SAH). This prospective case–control study included 43 patients with aneurysmal SAH and ventricular drainage for clinical reasons. Longitudinal CSF samples (99) were obtained in the course of SAH. Patients were dichotomized regarding the occurrence of cerebral vasospasm syndrome (CVS) (*N* = 19). Arginase-1 and the amino acids L-arginine and L-ornithine were quantified in CSF. Outcome assessments included delayed cerebral ischemia (DCI) and functional status after 3 months using the modified Rankin Scale (mRS). Arginase-1 was released into the CSF of SAH patients whereas this enzyme was undetectable in controls. Compared to patients without CVS, arginase-1 levels were higher in CVS patients until day 14 after clinical event. The well-known surrogate parameter for arginase acitivity, the L-arginine to L-ornithine ratio (Arg/Orn), correlated with CSF arginase-1 levels. Arg/Orn was reduced in patients with CVS from disease onset (days 1–3, *p* = 0.0009) until day 14. Logistic regression analysis of early Arg/Orn was predictive for CVS (*p* = 0.008) and DCI (*p* = 0.035), independent of age, Hunt and Hess grade, and intraventricular blood. Arg/Orn < 2.71 at disease onset predicted CVS with a sensitivity of 86.7% and specificity of 72.2%. Arg/Orn ≥ 2.71 predicted excellent functional outcome. We propose a novel mechanism contributing to NO deprivation during SAH: arginase-1 is released from erythrocytes into the CSF, leading to L-arginine consumption and reduced NO bioavailability. Furthermore, Arg/Orn is a robust predictor for occurrence of CVS, DCI, and functional outcome 3 months after aneurysmal SAH. Our data provide a novel prognostic biomarker and may contribute to the development of novel therapeutic strategies in SAH. Clinical Trial Registration-URL: http://www.drks.de. Unique identifier: DRKS00015293, date of registration: 13.09.2018.

**Supplementary Information:**

The online version contains supplementary material available at 10.1007/s12975-021-00944-y.

## Introduction

Despite improved surgical and endovascular treatment options for ruptured intracranial aneurysms, up to 70% of subarachnoid hemorrhage (SAH) patients develop spasms of cerebral arteries [1]. These vasospasms as well as impaired microcirculation contribute to delayed cerebral ischemia (DCI) leading to permanent neurological deficits or death in half of the patients [2, 3]. Though its major impact on patient outcome, the pathophysiology of DCI remains elusive [4]. It is widely accepted that intracellular products released from lysed erythrocytes to the subarachnoid space are responsible for the development of cerebral vasospasm syndrome (CVS) and DCI [5]. Due to its 1000-fold higher affinity for nitric oxide (NO) than oxygen, hemoglobin and its catabolites are suspected of scavenging NO [6]. NO regulates cerebral blood flow by its endothelial dilatory capacity and decreased NO levels closely correlate with the degree of DCI [7]. Furthermore, treatment with NO or NO-donors prevents and reverses vasospasm after SAH [8].

Under physiological conditions, NO is synthesized mainly by the endothelial nitric oxide synthetase (eNOS) from the amino acid L-arginine [9]. NO is cleaved from the terminal guanidino nitrogen atom of L-arginine by NOS, producing L-citrulline. Competitively, L-arginine is catabolized by the enzyme arginase into L-ornithine and urea as the final step of the urea cycle [10]. Two isoforms of arginase exist; arginase-1 is expressed predominantly in the liver, but is also present in the cytoplasm of erythrocytes [11]. Arginase-2 is a mitochondrial enzyme found primarily in kidney and prostate [12]. In several hemolytic disorders like sickle cell disease, thalassemia, and paroxysmal hemolytic ureamia, arginase-1 is released from erythrocytes into the plasma causing NO depletion and secondary pulmonary hypertension [13–16]. In these disorders, plasma arginase-1 levels correlate with markers of hemolysis like cell-free hemoglobin [13].

We hypothesized that during aneurysmal SAH arginase-1 is released into the cerebrospinal fluid (CSF), leading to a dysregulation of L-arginine and NO metabolism. By depletion of the eNOS substrate L-arginine, arginase-1 may contribute to CVS, DCI and poor clinical outcome.

## Materials and Methods

### Patients

This prospective, non-interventional clinical trial was performed in a single tertiary university hospital. It was registered in the German Clinical Trials Register (DRKS-ID: DRKS00015293) and approved by the local ethics committee. The study was carried out according to the guidelines of Helsinki declaration and patients/guardians/relatives signed informed consent.

Inclusion criteria were as follows: (1) age ≥ 18 years, (2) aneurysmal SAH with Fisher grade of 3 including patients with and without intraventricular hemorrhage (IVH), (3) ventricular drainage insertion indicated by the treating clinician to monitor intracranial pressure in patients with vigilance reduction on admission or during the course of the disease, in a few cases due to hydrocephalus. Exclusion criteria were mycotic aneurysms, known chronic infections or hemophilia, and missing relatives if patients were unable to give their informed consent.

### Clinical Monitoring, Treatment, and Clinical Outcome

We followed our standardized diagnostic and treatment regimen. Hunt and Hess graduation was performed upon hospital admission and SAH was proven by CT scan. CT angiography (CT-A) and digital subtraction angiography (DSA) were performed for further evaluation of the aneurysm. The treatment decision (coiling or clipping) was based on an interdisciplinary approach. We followed an early treatment strategy within 48 h of admission. All patients with aneurysmal SAH received nimodipine from the day of clinical admission. Screening for cerebral vasospasm was performed daily using neurological examination and transcranial Doppler ultrasound (TCD) measurements as described earlier [17]. If vasospasm was suspected on the basis of TCD or delayed ischemic neurological deficit (DIND), CT-angiography/-perfusion (CT-A, CT-P) were performed in order to confirm cerebral vasospasm. Presence of clinically relevant cerebral vasospasm syndrome (CVS) was defined as vasospasm-associated DIND and/or vasospasm-related perfusion deficit in CT-P and/or moderate to severe narrowing of intracranial arteries compared to the original size. In cases of onset of clinical-relevant CVS, hypertension was induced with catecholamines during treatment course. Delayed cerebral infarctions (DCI) were defined as occurrence of new ischemic lesions on any radiological imaging that was absent on admission or up to 24 h postoperatively and could not be attributed to other causes [18]. Functional outcome was assessed 3 months after SAH using the modified Rankin scale (mRS). mRS scores 0 and 1 were defined as excellent functional outcome, mRS scores 2 to 3 as moderate clinical outcome, 4 to 5 as poor clinical outcome, and mRS score 6 as dead.

### Sample Collection and Preparation

CSF samples were collected repetitively every 2–4 days from the time of ventricular drainage insertion for up to 22 days for measurement of protein levels and up to 14 days for analysis of amino acid levels, or until removal of the ventricular drainage by discretion of the treating physician, whatever occurred first. Samples were centrifuged immediately and stored at − 80 °C until further analysis. Control CSF samples were collected of 21 patients without SAH, but requiring spinal tap due to primary headache or peripheral facial palsy, and processed equally.

### Biochemical Analysis

Arginase-1 was measured in CSF at a 1:4 dilution using ELISA (Hycult biotechnology) according to the manufacturers’ protocol. Lower detection level was 6.4 ng/ml. All samples were measured in duplicates. The absorbance was measured at 450 nm. L-arginine and L-ornithine were measured in certified clinical laboratories using high-performance liquid chromatography (HPLC) or liquid chromatography tandem mass spectrometry (LC–MS/MS). Both methods were commercially available for the determination of the amino acid profile in CSF samples without modification.

### Sample Grouping and Statistical Analysis

CSF-samples were grouped into 4 different time points after SAH: pre-CVS phase (days 1–3, e.g., 24–72 h after SAH), CVS onset phase (days 4–7, e.g., 73–168 h after SAH), manifest CVS phase (days 8–14), and CVS remission phase (days 15–22). If more than one sample from a patient was available at a single time point, mean values were calculated. Clinical data including demographic data, CVS, arginase-1 concentration, and L-arginine/L-ornithine ratio were analyzed by Student’s *t* test for parametric data and Mann–Whitney *U* test or Fisher´s exact test for ordinal data. For correlation analysis between arginase-1 concentration and L-arginine/L-ornithine ratio, Spearman’s correlation analysis was applied. Diagnostic performance was evaluated using receiver-operating-characteristic (ROC) analyses. Logistic regression analyses were performed for prediction of outcome and CVS/DCI. Statistical significance was accepted at an alpha level of *p* < 0.05 and all analyses were two-sided. Statistical analyses and graphing were performed with GraphPad Prism (GraphPad Software Inc.) or SPSS (Version 25, IBM, Armonk, NY).

## Results

### Clinical Data

Of 102 patients treated for aneurysmal SAH during the study period, 43 patients were enrolled. All received a ventricular drainage for treatment of hydrocephalus or intracerebral pressure monitoring. Patients were rated Hunt and Hess grade I in 4 cases, grade II in 12 cases, grade III 7 cases, grade IV in 7 cases, and grade V in 13 cases. Aneurysms were secured by clipping (40%) or coiling (60%). Forty-four percent of patients (19/43) developed CVS during the course of disease and 28% (12/43) DCI. Meningitis or ventriculitis was diagnosed in 14% of patients, which was detected at earliest after 9 days. IVH, detected within 72 h after SAH, was more frequent in patients with CVS (*p* = 0.02). Other baseline clinical characteristics were balanced between patients with and without CVS (Table 1). Hunt and Hess grade was numerically higher in CVS patients without reaching statistical significance (*p* = 0.35). Consistently, CVS was significantly associated with DCI (*p* < 0.0001) and worse clinical outcome after 3 months (*p* = 0.04). In 33 patients, CSF was available within the first 3 days after SAH and 99 CSF samples were retrieved in total. Availability of CSF samples was distributed equally between groups. Within the first 2 weeks of the study, 3 patients passed away with 2 suffering from CVS.

**Table 1:** Patient sample characteristics

	all SAH patients (n = 43)	CVS (n = 19)	no CVS (n = 24)	p-value
Sex, female (%)	24 (56%)	12 (63%)	12 (50%)	*.54*
Age, mean (SD), years	59.1 (± 11.2)	58.3 (± 8.3)	59.7 (± 13.2)	*.69*
H&H, median (IQR)	3 (2–5)	4 (3–5)	3 (2–4)	*.33*
Intervention: Clipping (%)*	17 (40%)	9 (47%)	8 (33%)	*.53*
IVH, present (%)	14 (33%)	10 (53%)	4 (17%)	***.02***
DCI, present (%)	12 (28%)	12 (63%)	0 (0%)	***< .0001***
Meningitis, present (%)	6 (14%)	2 (10%)	4 (17%)	*.68*
outcome mRS, median (IQR)	3.5 (1.0–5.0)	4.0 (1.75–5.0)	1.0 (1.0–5.0)	***.04***

*SAH* subarachnoid hemorrhage, *CVS* cerebral vasospasm syndrome, *SD* standard deviation, *H&H* Hunt and Hess grade, *IQR* interquartile range, *IVH* intraventricular hemorrhage visible within 24 h after SAH, *DCI* delayed cerebral infarction, *mRS* modified Rankin scale, *all other cases underwent endovascular coiling

### Arginase-1 Is Released into the CSF Early After SAH and Predicts CVS

**Fig. 1:**
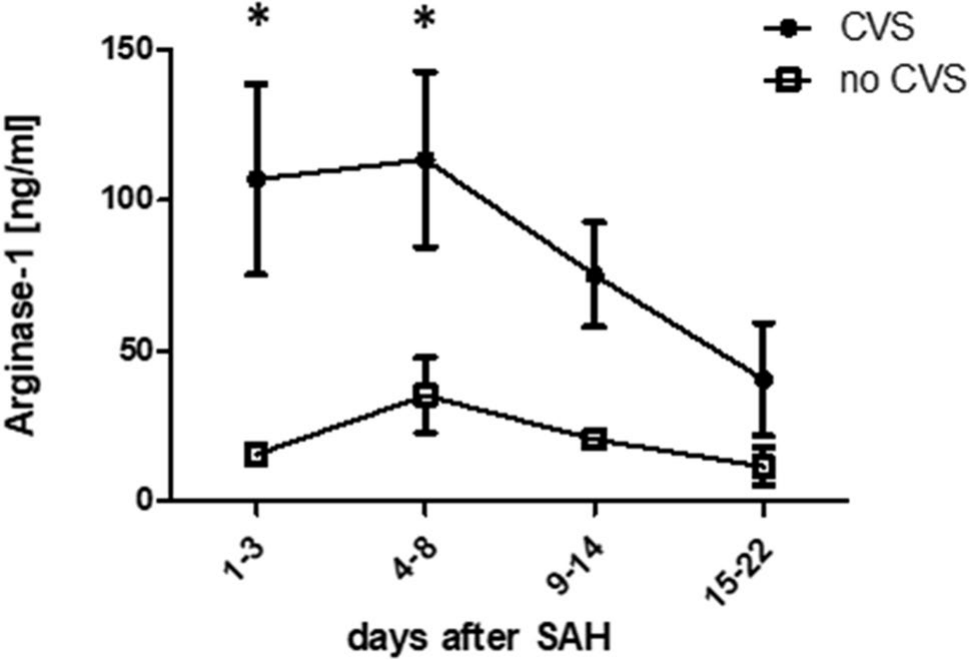
Arginase-1 is released into CSF after SAH. In SAH-patients developing CVS after aneurysmal SAH, arginase-1 levels are significantly higher compared to patients without CVS during pre-CVS phase (*n* = 9 vs. 5) and at CVS onset phase (*n* = 11 vs. 6). Altogether, arginase-1 concentrations peak between days 4 and 8 when clinical CVS occurs (*p < 0.05). In controls, Arginase-1 levels were below detection limits in CSF

Ninety percent of CSF samples from SAH-patients contained arginase-1, while arginase-1 levels were below detection limits in CSF from control patients (*p* = 0.0001, *n* = 23 vs. 5, data not shown). In SAH patients, arginase-1 levels peaked at 4–8 days, returning to lower levels between days 15 and 22 (Fig. 1). Arginase-1 levels were significantly higher in patients developing CVS during the course of disease compared to patients without CVS both during pre-CVS phase (*p* = 0.029, *n* = 9 vs. 5) and CVS onset phase (*p* = 0.038, *n* = 11 vs. 6). At later time points, this numerical difference lost statistical significance. Overall, CSF arginase-1 concentration was highly variable.

### L-Arginine/L-Ornithine Ratio Is Reduced in CSF After SAH in Patients with CVS

**Fig. 2:**
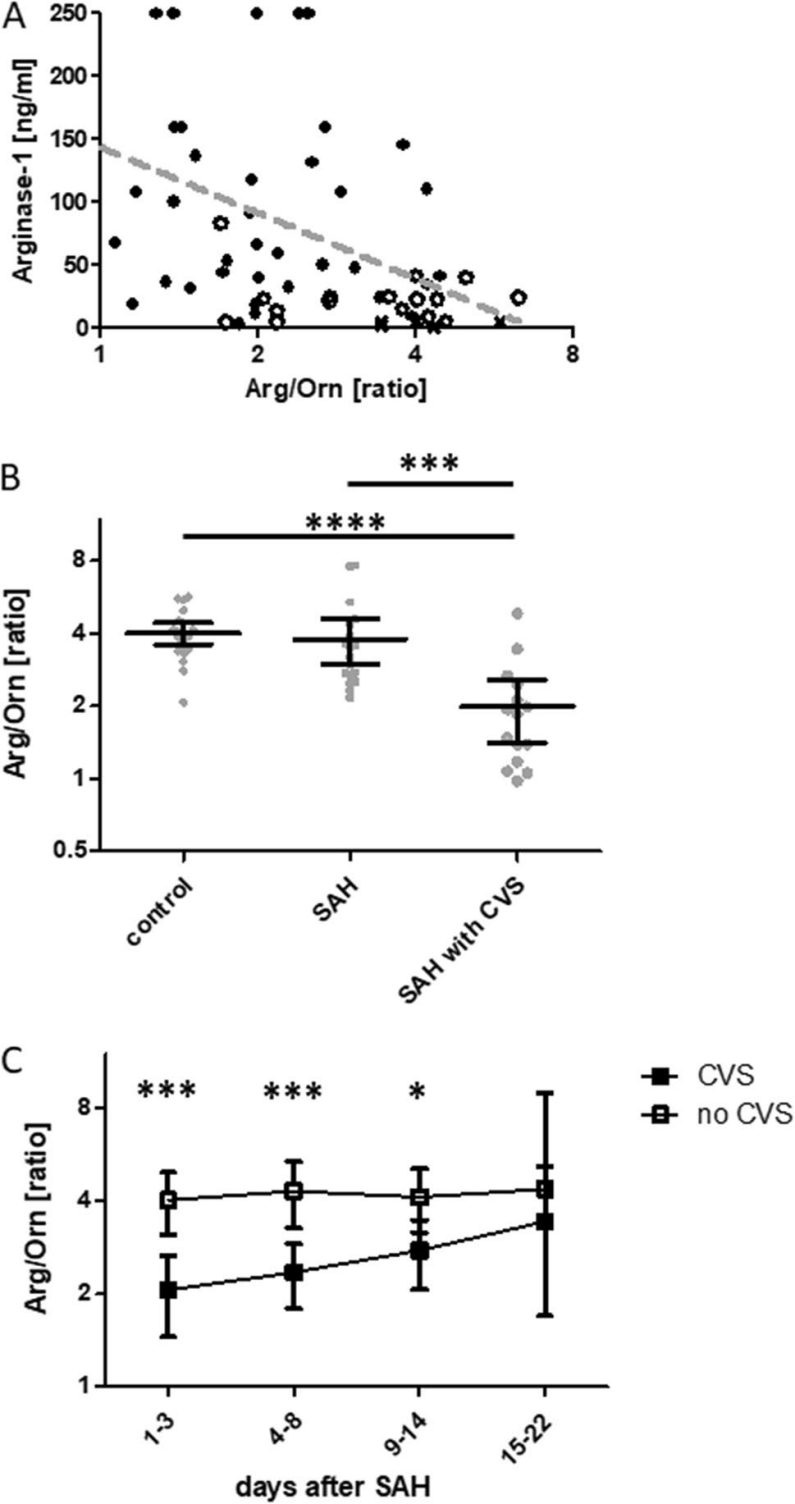
CSF Arg/Orn negatively correlates with Arginase-1 concentrations and is reduced in patients with CVS early after SAH*.* **A** Spearman correlation of CSF Arginase-1 concentrations and Arg/Orn and SAH patients with CVS (●), without (○), and controls (×) (*n* = 60). **B** Arg/Orn in controls (*n* = 21), SAH patients without CVS (*n* = 18), and with CVS (*n* = 15) obtained within 24 to 72 h after bleeding event (***p < 0.001, ****p < 0.0001). **C** Longitudinal CSF Arg/Orn in SAH patients with or without CVS. Pre-CVS phase (*n* = 15 vs 18), CVS onset phase (*n* = 15 vs. 17), manifest CVS phase (*n* = 13 vs. 12), and CVS remission phase (*n* = 6 vs 3) (***p < 0.001, *p < 0.05). All plots are mean ± 95% CI

To investigate arginase-1 activity in CSF, we analyzed its substrate L-arginine and its metabolite L-ornithine. The L-arginine/L-ornithine ratio (Arg/Orn) is well-established as an indirect measure of arginase activity in hemolytic diseases, e.g., sickle cell disease [13]. Arg/Orn was remarkably stable in both SAH patients and controls and negatively correlated with arginine-1 levels (spearman’s *r* =  − 0.46, *p* = 0.0002, *n* = 60) (Fig. 2).

Arg/Orn was similar between control patients and SAH patients without CVS (Fig. 2B). However, patients developing CVS later in the course of disease exhibited a significantly lower Arg/Orn at the pre-CVS phase compared to both controls (*p* < 0.0001, *n* = 22 vs. 15) and SAH patients without CVS (*p* = 0.0009, *n* = 18 vs. 15). Interestingly, modality of treatment did not influence Arg/Orn (mean Arg/Orn clipping: 2.60 ± 0.32, vs. coiling: 3.17 ± 0.41, *p* = 0.35, data not shown).

Longitudinal analysis also confirmed significantly lower values of Arg/Orn in CVS patients than patients without CVS at later time points during the first 2 weeks of disease (vasospasm onset phase: *p* = 0.0010; early vasospasm phase: *p* = 0.0101). While Arg/Orn was stable in patients without CVS, it gradually increased in CVS patients, until the group difference lost statistical significance from day 15 on (Fig. 2C).

### Arg/Orn in CSF Is Predictive for CVS and DCI in SAH

Arg/Orn was predictive of CVS in logistic regression analysis with an OR of 4.36 (95% CI: 1.47–12.87, *p* = 0.008) at pre-CVS time point and with an OR of 2.41 (95% CI: 1.24–4.68, *p* = 0.010) at vasospasm onset time point per decrease of Arg/Orn by 1. These results were confirmed in multivariate logistic regression analysis correcting for Hunt and Hess grade, age, and IVH (Table 2). Arg/Orn at both pre-CVS and vasospasm onset time point also predicted DCI (OR 2.9, 95% CI: 1.08–7.80, *p* = 0.035 and OR 2.40, 95% CI: 1.08–5.29, *p* = 0.030, respectively).

**Table 2:** Multivariable logistic regression analysis for the predictive value of Arg/Orn on DCI (A: pre-CVS time point, B: CVS onset time point)

A		*OR*	*95% CI*	*p-value*
	Arg/Orn (d1–3)	3.67	1.11–12.19	***.034***
	Hunt&Hess	1.23	0.53–2.87	*.63*
	Age	1.03	0.95–1.11	*.54*
	IVH	0.18	0.02–2.06	*.17*
B		*OR*	*95% CI*	*p-value*
	Arg/Orn (d4–8)	3.19	1.17–8.66	***.023***
	Hunt&Hess	0.58	0.52–3.22	*.58*
	Age	1.08	0.98–1.20	*.13*
	IVH	0.17	0.01–2.16	*.17*

*Arg/Orn* L-arginine to L-ornithine ratio, *IVH* intraventricular hemorrhage, *OR* odds ratio, *CI* confidence interval

**Fig. 3:**
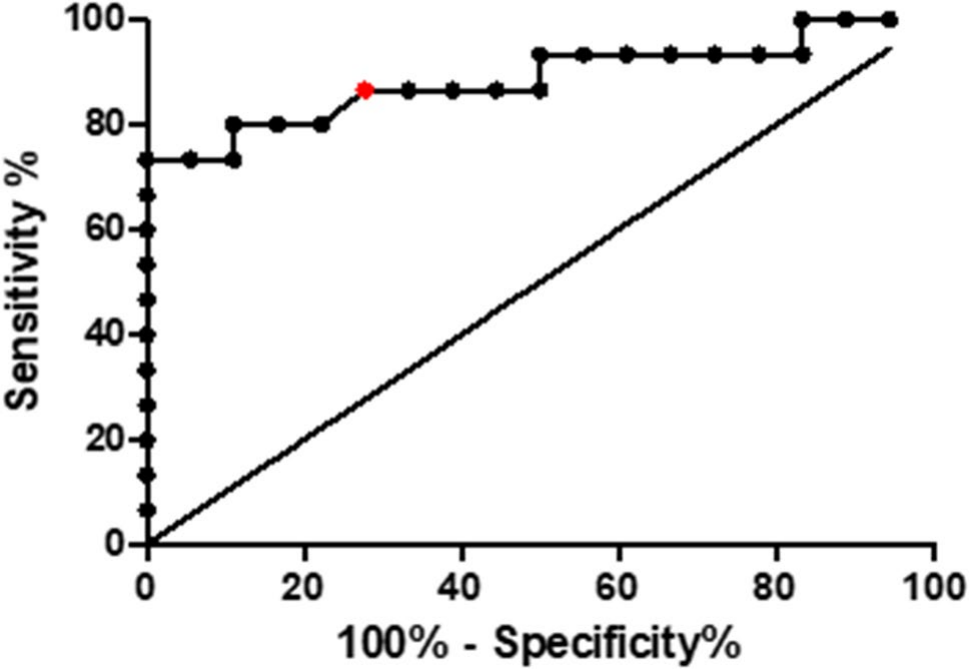
CSF Arg/Orn is predictive for CVS within 24–72 h after SAH. ROC analysis of Arg/Orn provided a ratio of 2.71 as optimal cutoff for prediction of CVS (red dot, sensitivity = 86.7%, specificity = 72.2%, accuracy = 79%)

Diagnostic performance of Arg/Orn for early prognostication of CVS on days 1–3 was evaluated using ROC analysis. Optimal Arg/Orn cutoff for prediction of CVS was 2.71 (sensitivity = 86.7%, specificity = 72.2%, accuracy = 79%, area under the curve = 0.887 ± 0.065, *p* = 0.00016, Fig. 3).

### Low CSF Arg/Orn at Disease Onset Predicts Functional Outcome at 3 Months

**Fig. 4:**
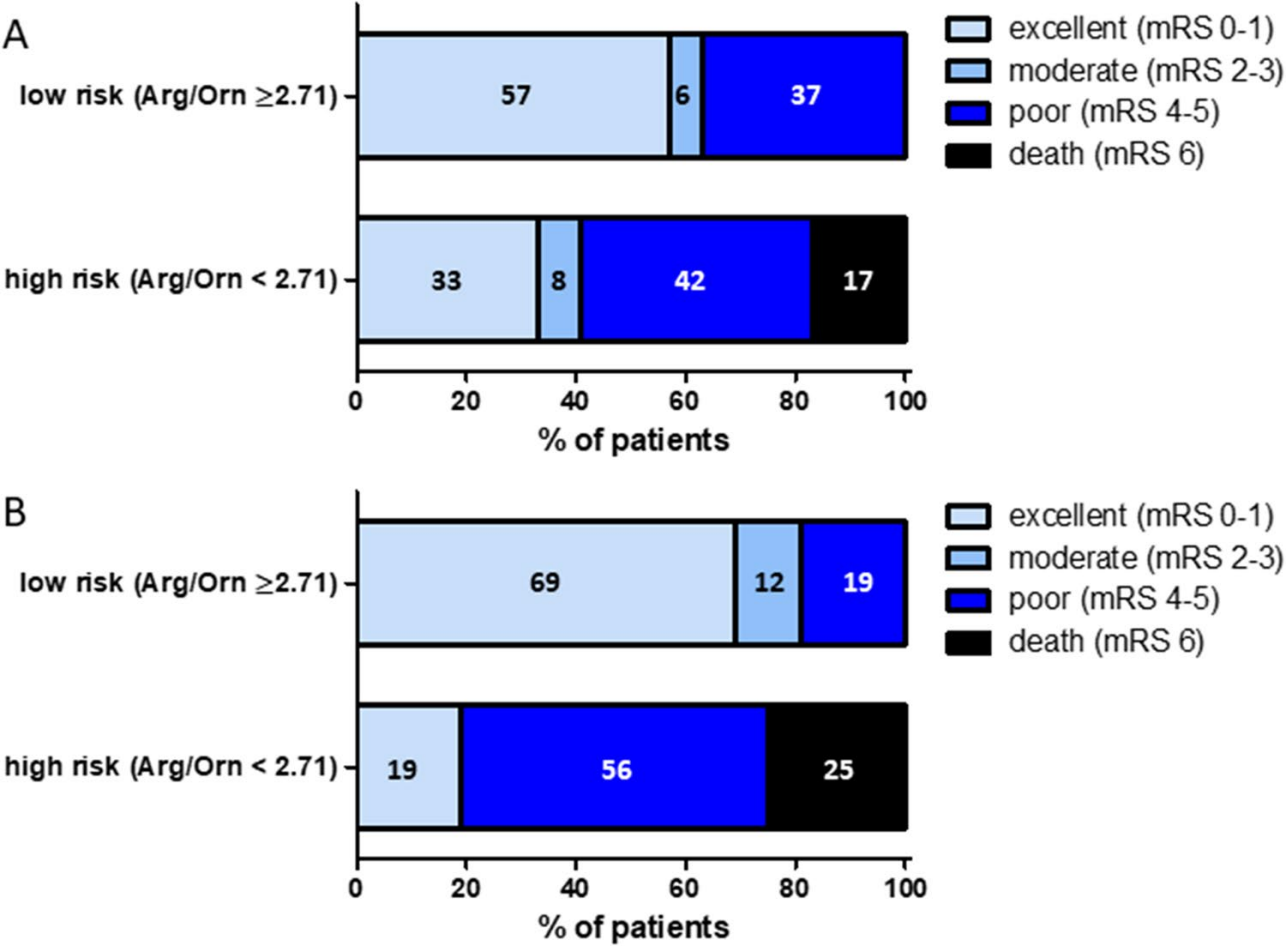
CSF Arg/Orn < 2.71 is predictive for worse functional outcome. Diagram showing association of functional outcomes 3 months after SAH with Arg/Orn < 2.71 at pre-CVS phase (**A**) and CVS onset phase (**B**). Each box of the horizontal bar represents a functional mRS category specified by the color code. Numbers in each box denote the percentage of patients belonging to a risk group stratified by Arg/Orn. If no patient met a specified outcome category, the box is missing

The Arg/Orn cutoff value of 2.71 was tested as a predictor of functional outcome 3 months after SAH. In logistic regression analysis, Arg/Orn ≥ 2.71 within the first 3 days after SAH was predictive of excellent functional outcome defined as mRS 0–1 with an OR of 4.88 (95% CI 1.06–22.38; *p* = 0.042). Prediction was similar in CSF samples drawn on days 4–7 with an OR of 6.67 (95% CI 1.28–33.33; *p* = 0.024). Intriguingly, death was only observed in SAH patients with Arg/Orn < 2.71 at these time points (Fig. 4). Ordinal logistic regression analysis confirmed a significant shift in clinical outcome for patients with Arg/Orn < 2.71 between days 1 and 8 after SAH (*n* = 39, *p* = 0.004).

## Discussion

The importance of NO dysregulation for the development of vasospasm and ischemic neurological deficits after SAH is generally accepted [4]. Erythrocytes and their degradation products have been shown to be the main driver of NO depletion and vasospasm [19, 20]. Furthermore, CSF concentration of asymmetric dimethylarginine (ADMA), a known inhibitor of NOS, has been shown to correlate with CVS in SAH [21]. In hemolytic disorders, such as sickle cell disease, thalassemia, or paroxysmal nocturnal hemoglobinuria, NO deficiency is caused by release of arginase-1 from erythrocytes, leading to consumption of L-arginine finally resulting in vasoconstriction and pulmonary hypertension [13, 16, 22, 23].

Based on these findings, we hypothesized that a similar mechanism may promote vasospasm after SAH: arginase-1 is released from lysed erythrocytes into the subarachnoid space, amplifying depletion of L-arginine and production of L-ornithine, thereby contributing to NO deficiency and vasospasm formation. Indeed, arginase-1 protein was detected in CSF after SAH, but not in control CSF samples. While CSF arginase-1 may also originate from leakage through the disrupted blood brain barrier during SAH [24], its time course argues for release from lysed erythrocytes [25, 26]. Intriguingly, SAH patients developing CVS during the course of disease exhibited higher CSF arginase-1 levels from day 1 on, peaking around day 7, when CVS onset is usually observed.

Unfortunately, measurement of arginase-1 is not yet a validated analytical procedure in commercial laboratories. Therefore, we investigated concentrations of amino-acids in CSF, which is available to most physicians. As L-arginine is metabolized by arginase-1, decreased L-arginine levels could be expected in these patients, but earlier studies were unable to find an association between L-arginine levels and vasospasms [27, 28]. This might be due to highly variable L-arginine levels in CSF, possibly attributable to varying amounts of subarachnoid blood or blood–brain-barrier leakage. Therefore, Arg/Orn, a robust parameter used in studies of hemolytic disorders, was calculated [13]. Arg/Orn is expected to correct for absolute levels of amino acids and might correlate even better with intracellular L-arginine deficiency, as L-ornithine and L-arginine compete for uptake via cationic amino acid transporters [29]. Indeed, Arg/Orn was reduced in SAH patients developing CVS and allowed prediction of CVS, DCI, and most importantly clinical outcome. Furthermore, the effect of Arg/Orn on the development of CVS was independent of established predictors for CVS (e.g., IVH [30]) in the present multivariable analysis. Because 3 patients passed away during the first 14 days of the study, the convergence of the Arg/Orn ratio in both groups over time may be due to censoring of individual values.

Based on these data, this trial provides a biomarker for occurrence of CVS, DCI, and worse clinical outcome after aneurysmal SAH, with Arg/Orn < 2.71 as a preliminary cutoff value. According to the standard reporting recommendations for biomarkers in aneurysmal subarachnoid hemorrhage studies [31], the following standard operating procedure is suggested: 24–72 h after SAH, a ventricular CSF sample should be obtained, centrifuged, and frozen, followed by amino acid profiling in an accredited clinical laboratory. Calculation of Arg/Orn allows prediction of CVS, DCI, and outcome. Reanalysis of Arg/Orn between 73 and 96 h after SAH may be performed [32] to reaffirm predictions (supplementary Fig. 1). However, larger multicentric studies are required to confirm these findings, refine cutoff values, and optimize sampling strategies.

Free hemoglobin and its degradation product Oxyhemoglobin (OxyHb) is probably a key driver of arterial narrowing in experimental models of SAH [33]. However, clinical evidence for a prognostic value of intrathecal OxyHb is controversial. Although intrathecal OxyHb and Arg/Orn possibly correlate, this study focused on amino acid analysis because this method is simple and readily available in most laboratories.

This study has several limitations. As this was a proof of principle study, we were not able to perform statistic power analysis before recruitment. Nevertheless, we achieved a clear statistical significance of our results. Lumbar CSF from patients with peripheral facial palsy or headache was used as control samples, but comparability of lumbar and ventricular CSF samples may be reduced. However, CSF from SAH patients with and without CVS was analyzed after identical preanalytical handling, rendering the main findings of this study independent from non-SAH controls. Patients with severe SAH were possibly overrepresented in this study, as insertion of a ventricular drain was an inclusion criterion and 13 cases of Hunt and Hess grade V SAH were included. This may explain the frequent occurrence of DCI in this study compared with larger cohorts [34]. Further studies should therefore also evaluate CSF samples from spinal tap or lumbar CSF drainage to include patients with less severe SAH. Cerebral microdialysis might be a valuable means for repeated measurement of Arg/Orn.

Arginine is a substrate for numerous other metabolic pathways such as decarboxylation to agmatine, the cleavage of NO by NOS, or a basic component of protein synthesis [35, 36]. Therefore, the Arg/Orn ratio cannot provide a complete measure of arginine metabolism, but it is an established biomarker for quantifying arginase activity [37]. Another established ratio for Arginine bioavailability, defined as Arginine/(Ornithine + Citrulline) ratio, was not applicable in this study because citrulline in CSF is below the limit of detection.

As this is a single-center study, number of subjects is low preventing to account more variables in logistic regression analysis like securing of aneurysm or aneurysm size. As validation in an independent cohort is needed, we searched the literature for longitudinal reports of L-arginine and L-ornithine in CSF of SAH patients and identified two relevant clinical trials [38, 39]. In accordance to our results, Arg/Orn as estimated from reported values was decreased in SAH patients compared to healthy controls (0.97–2.55 vs. 3.65–4.18, respectively). Intriguingly, Sokół et al. reported significantly increased L-ornithine levels in patients with poor clinical outcome [39]. Estimated Arg/Orn based on reported median values was lower in SAH patients with poor outcome, defined as persistent vegetative state or death, compared to patients with good outcome (0.81 vs. 2.43, respectively). Moreover, a recent metabolomic investigation reported an association between elevated L-ornithine concentrations and poor patient outcome [40]. These reports confirm the association between Arg/Orn and clinical outcome in SAH.

In patients suffering from intracerebral hemorrhage, early reduction in CSF L-arginine concentration was an independent risk factor for poor outcome, comparable to our study [41]. Microvascular dysfunction leading to secondary brain damage is well described after intracerebral hemorrhage and might follow similar mechanisms like in SAH. We speculate that the local distribution of arginase-1 and L-arginine depletion near the cerebral basal arteries contributes to the frequent occurrence of generalized CVS after SAH. In patients with EVD after tumor resection or traumatic brain injury, exemplary Arg/Orn samples showed comparable levels to healthy controls. Inconsistent results were seen in patients with intracerebral hemorrhage and ventricular blood clots, comparable to the study by Mader et al. [41].

The proposed pathophysiological mechanism has implications for possible therapeutic strategies in SAH. Firstly, our study provides a rationale to study therapeutic inhibition of arginase-1 in SAH-patients. Though Arg/Orn is prognostic for long-term clinical outcome already early after disease onset, we see a window of therapeutic opportunity by blockade of arginase and/or normalization of Arg/Orn. Still 40% of patients with low Arg/Orn between 24 and 72 h have a favorable outcome, whereas this proportion decreases to 20% around 4 days after SAH. There are several specific arginase-1 inhibitors available like nor-N-hydroxy-L-arginine (nor-NOHA), 2(S)-amino-6-boronohexanoic acid (ABH), and S-(2-boronoethyl)-L-cysteine (BEC) [42]. Furthermore, the compounds INCB001158 and CB-280 with arginase inhibiting properties are under advanced clinical investigation for indications like tumor treatment and cystic fibrosis. INCB001158 is able to induce a threefold increase in plasma arginine levels at therapeutic doses with a reasonable safety profile. Systemic or intraventricular administration of these inhibitors warrants testing in experimental models of SAH and clinical trials. Additionally, restoration of Arg/Orn by intrathecal application of L-arginine could be a promising approach to prevent CVS and DCI and improve clinical outcome in SAH patients with reduced Arg/Orn. Indeed, intracisternal or intraperitoneal injection of L-arginine reversed experimental vasospasm in dogs [43] and rats [44]. Intracarotid infusion of L-arginine led to a markedly increased regional cerebral blood flow, but did not influence the incidence of CVS in a primate model of SAH [45], possibly due to the short half-life of L-arginine. Therefore, a continuous intraventricular infusion could be necessary and should be evaluated in future animal studies.

## Summary/Conclusion

This study presents Arg/Orn in CSF as a concise, robust, largely available, and pathophysiologically plausible parameter for prediction of CVS, DCI, and clinical outcome in aneurysmal SAH. We propose that arginase-1 released from erythrocytes into the subarachnoid space modulates Arg/Orn, possibly assuming a causative role in the reduction of NO bioavailability responsible for occurrence of ischemic brain injury. These findings might lead to improved prognostication and novel therapeutic possibilities in treatment of subarachnoid hemorrhage.

## Supplementary Information

Below is the link to the electronic supplementary material.

Supplementary file1 (PDF 122 KB)

## Data Availability

All authors declare that all data support our published claims and comply with field standards. The data are available from the corresponding author upon reasonable request.
