# Supplementary material for: Arginase-1 Released into CSF After Aneurysmal Subarachnoid Hemorrhage Decreases Arginine/Ornithine Ratio: a Novel Prognostic Biomarker
Source: Transl Stroke Res. 2021 Oct 2;13(3):382–90. doi: 10.1007/s12975-021-00944-y (PMC9046143; doi:10.1007/s12975-021-00944-y)
Supplement: Supplementary file 1 — Supplementary file1 (PDF 122 KB) [file 12975_2021_944_MOESM1_ESM.pdf]

SOP: Arg/Orn for risk stratification after SAH

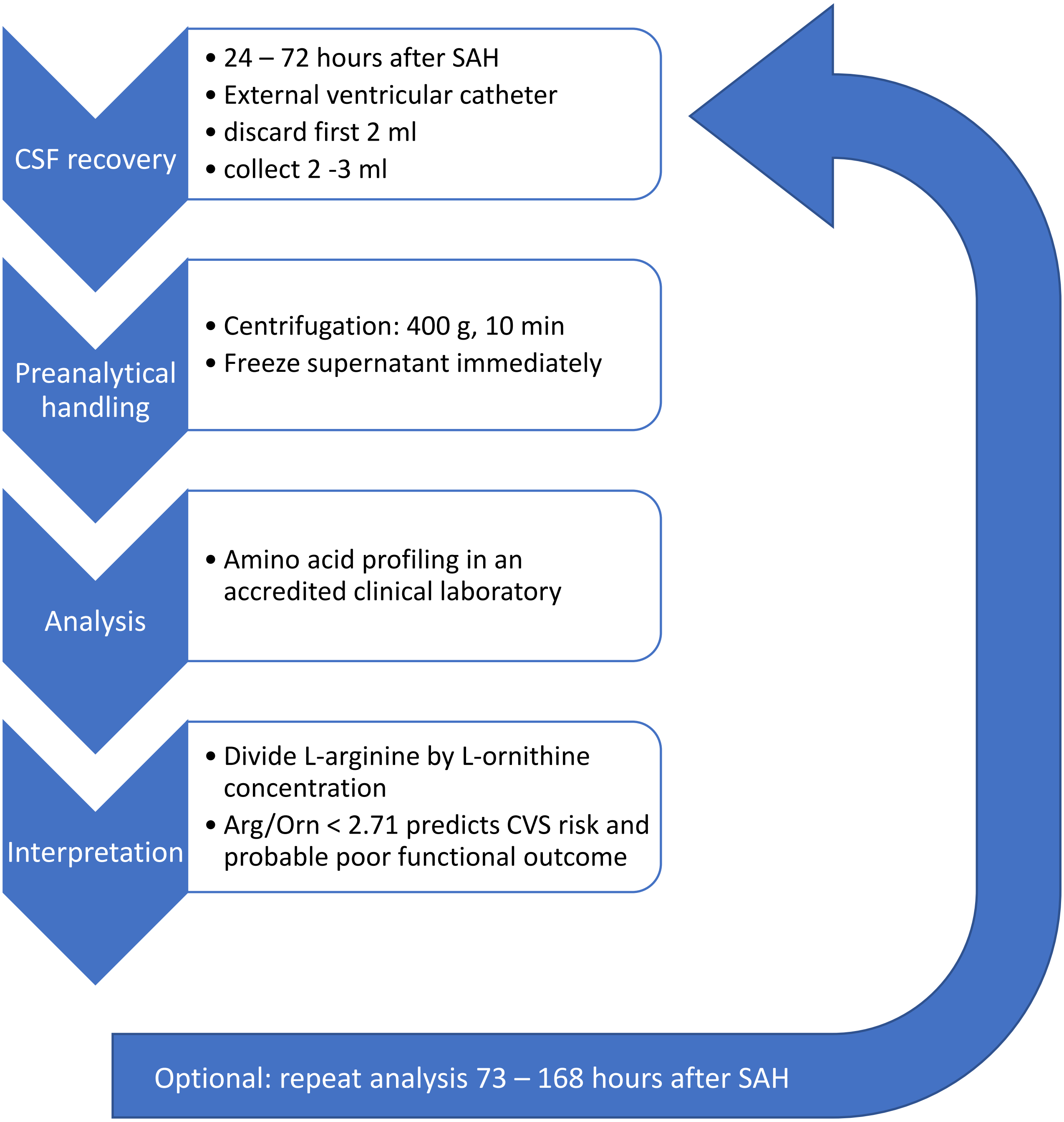

Supplementary Figure I: Proposals for a preliminary standard operating procedure (SOP) stratifying CVS-risk and clinical outcome according to the standard reporting recommendations for biomarkers in aneurysmal subarachnoid hemorrhage studies[30].

SOP: standard operating procedure, Arg/Orn: L-Arginine/L-Ornithine ratio, SAH: subarachnoid hemorrhage, min: minute, CVS: cerebral vasospasm syndrome
